# The role of the microbiome on immune homeostasis of the host nervous system

**DOI:** 10.3389/fimmu.2025.1609960

**Published:** 2025-07-31

**Authors:** Shaojuan Zhao, Danlei Fu, Yin Lin, Xiaoya Sun, Xiaokang Wang, Xuanzhen Wu, Xuejiao Zhang

**Affiliations:** ^1^ Shenzhen Futian Third People’s Hospital, Shenzhen, China; ^2^ Medical School, Shenzhen University, Shenzhen, China; ^3^ Shenzhen Longhua District Central Hospital, Shenzhen, China

**Keywords:** gut microbiota, microglia, microbiota-gut-brain axis, aromatic compounds, neuroinflammation

## Abstract

The gut microbiota is often termed the “second genome” of the human body. It has been shown to be one of the most significant environmental factors (non-genetic) influencing the onset, progression, and prognosis of various neurological and psychiatric disorders through its interactions with the host immune, nervous, and endocrine systems. Changes in the function and composition of the gut microbiota are strongly associated with amyotrophic lateral sclerosis, autism spectrum disorder, depression, Parkinson’s disease, and Alzheimer’s disease. This review summarizes the research regarding the associations and regulatory mechanisms between the gut microbiota and the central nervous system in order to explore the role of the gut microbiota in maintaining neural homeostasis.

## 1 Introduction

The mammalian gastrointestinal tract harbors trillions of diverse microorganisms, including fungi, viruses, and bacteria, collectively referred to as the gut microbiota (1). The luminal and mucosal gut microbiota and their derived products interact with the host intestinal barrier and immune cells to maintain intestinal homeostasis (2). In addition, the gut microbiome is essential for host immune function (3), influencing both peripheral and central nervous system (CNS) immune homeostasis *via* microbial components (4) and metabolites like lipopolysaccharides (LPS) (5), tryptophan metabolism derivatives (6), and short-chain fatty acids (SCFAs) (7). This review examines this “gut-neuroimmune axis”, highlighting the mechanisms through which modulation of this axis may impact host neurological disorders.

## 2 Structures involved in regulating neuroimmune responses in the gut-brain axis

### 2.1 Microglia

**Figure 1:**
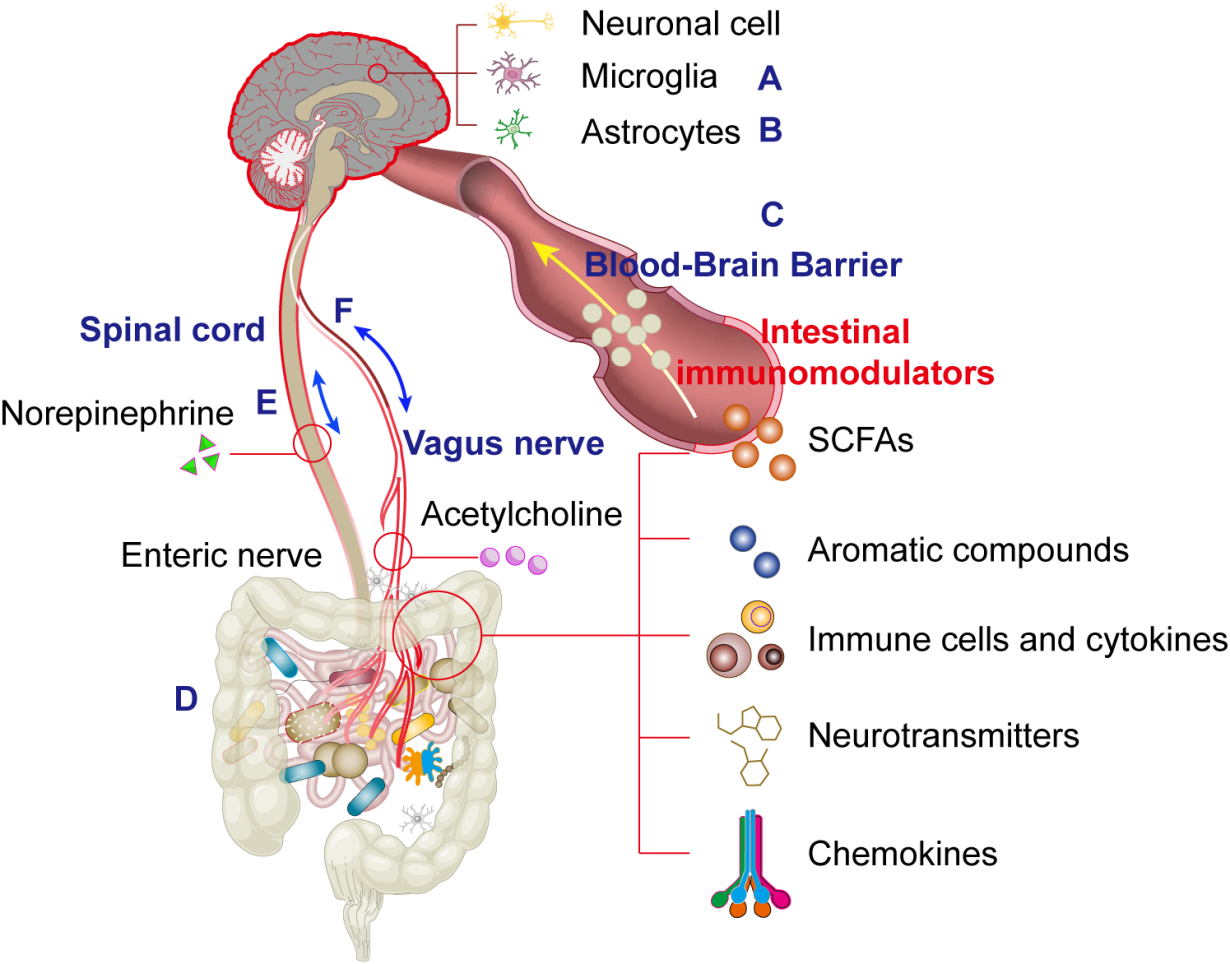
This schematic systematically illustrates the physiological structural basis of the microbiota-gut-brain axis (MGBA) mediated by bidirectional multi-channel communication, where interactions among microglia **(A)**, astrocytes **(B)**, the blood-brain barrier **(C)**, gut microbiota **(D)**, spinal cord **(E)** and the vagus nerve exhibits a dual-directional (bipolar) regulatory mechanism **(F)**.

Microglia are the resident myeloid cells of the CNS, comprising ~5-12% of brain cells (8). They are essential for maintaining brain homeostasis via neurogenesis, neurotransmission, synaptic remodeling, neuroinflammation, and injury repair (9). Microglia play a pivotal role in neuroinflammation by producing diverse molecular initiators and mediators (**Figure 1A**). They participate in neuroinflammatory events, regulate neural patterning, and mediate synaptic pruning (10). Upon activation, microglia release chemokines, cytokines, and antigenic markers, modulate neurotransmitter production and release, and undergo morphological changes (11). They maintain equilibrium by activating phenotypic responses and releasing both pro- and anti-inflammatory cytokines.

Under healthy conditions, microglia remain in an immunologically quiescent resting state due to inhibitory signals from cell-surface receptors and soluble ligands derived from surrounding neurons (12). During this state, microglia exhibit a highly branched morphology and they contribute to brain homeostasis by eliminating or remodeling synapses (13), supporting myelin renewal, monitoring neural activity, and actively clearing pathogens or localized tissue damage (14). Upon detecting brain injury, microglia undergo microglial activation and retract their processes, transitioning to an amoeboid morphology (15). It is widely accepted that microglial activation is initiated by the removal of inhibitory neuronal signals and the engagement of pattern recognition receptors (PRRs) by exogenous pathogen-associated molecular patterns (PAMPs) and/or endogenous damage-associated molecular patterns (DAMPs).

Activated microglia can adopt diverse and complex phenotypes, displaying distinct cell-surface and intracellular markers, secreting different cytokines, and performing specialized functions (16). These encompass the pro-inflammatory M1 phenotype and the anti-inflammatory M2 phenotype, which is further divided into M2a, M2b, and M2c subtypes. The early response following injury is characterized by pro-inflammatory activity driven by M1-polarized microglia (17). Eventually, M2-polarized microglia initiate an anti-inflammatory response crucial for tissue repair and a return to homeostasis. In contrast, microglia remain activated in an M1 phenotype by persistent pro-inflammatory stimuli during the chronic neuroinflammation characteristic of numerous neurodegenerative diseases (18). Persistently active microglia produce inflammatory cytokines and reactive oxygen/nitrogen species, leading to neuronal death (19).

### 2.2 Astrocytes

Astrocytes are tissue-resident stromal cells in the CNS that promote normal brain development and function by providing structural support, metabolite synthesis, neurotransmission regulation, and assisting in immune-related activities (20) (**Figure 1B**). Under physiological conditions, astrocytes also perform critical roles in pH, ion, and redox buffering, as well as regulating blood flow, neurotransmitter recycling, and energy homeostasis in the CNS. In pathological states, astrocytes undergo morphological and molecular changes, transitioning to a reactive state. During CNS inflammation and neurodegeneration, the cellular state of astrocytes correlates with the activation or suppression of specific genomic modules in response to disease-specific stimuli. For example, single-cell RNA sequencing (scRNA-seq) combined with proteomics has identified that under homeostatic conditions, astrocytes express tumor necrosis factor-related apoptosis-inducing ligand (TRAIL) in response to interferon-γ (IFN-γ) produced by natural killer (NK) cells. These astrocytes limit inflammation at CNS borders by inducing T-cell apoptosis.

In the CNS, microglia and infiltrating peripheral immune cells, such as T cells, primarily mediate inflammatory responses. However, macroglia, including astrocytes, serve as critical downstream effectors. Astrocytes can react to immunomodulatory cytokines and influence microglial activity by releasing pro- or anti-inflammatory cytokines (21). The crosstalk between astrocytes and microglia is important in maintaining CNS homeostasis. For example, it has been shown that astrocyte-derived IL-33 can facilitate microglia-mediated synaptic pruning during development, underscoring the critical role of astrocyte-microglia interactions in neural circuit formation (22). Conversely, microglia-derived cytokines influence pathogenic astrocyte functions in CNS inflammation. For example, Bezzi et al. showed that TNF-α from microglia triggered SDF-1-CXCR4 signaling in astrocytes, leading to the secretion of glutamate and causing neuronal death. Additionally, microglial-derived TNF-α, IL-1α, and C1q are known to trigger a neurotoxic astrocyte phenotype, and microglial-derived VEGF-B and TGF-α distinctly modulate pro-inflammatory gene expression in astrocytes during EAE and MS. Microglial VEGF-B amplifies pathogenic astrocyte activity in EAE by facilitating NF-κB activation through the VEGF receptor 1 (FLT-1), while microglial TGF-α mitigates EAE progression through the activation of ErbB1 signaling. Astrocytes also respond to microbiome-modulated systemic and central processes. Dietary tryptophan metabolites cross the blood-brain barrier, influencing aryl hydrocarbon receptor signaling in microglia and astrocytes, thereby regulating VEGF-B and TGF-α production and suggesting a novel mechanism through which the gut-brain axis regulates cells of the CNS. Another metabolite, D-β-hydroxybutyrate suppresses microglial activation, reducing IL-6 and TNF-α production and mitigating neuroinflammation (23). Similarly, indole-3-propionic acid (IPA) modulates TNF-α levels in activated microglia while supporting neuronal function (24).

The role of astrocytes in secreting chemokines to recruit lymphocytes is noteworthy. Research indicates that astrocytes generate chemokines like CXCL10, CXCL12, and CCL2, which play a crucial role in guiding immune cells to sites of inflammation (25). For example, CXCL10^+^ astrocytes are observed perivascularly in MS patients, suggesting a role in recruiting lymphocytes to the CNS and underscoring the potential for astrocytes to modulate disease progression via chemokine secretion. The influence of astrocytes on T-cell responses occurs through the release of cytokines like interleukin (IL)-6, IL-2, and TGF-β, which play pivotal roles in coordinating immune responses and regulating neuroinflammation. For instance, IL-6 exacerbates CNS autoimmunity, while IL-2 is essential for the expansion of neuroprotective regulatory T cells (Treg).

Neurodegenerative diseases, including Alzheimer’s disease, Parkinson’s disease, and amyotrophic lateral sclerosis contribute to inflammation in the CNS, although their underlying mechanisms are not fully understood. Compelling data suggest that in some neurodegenerative disorders, initial pro-inflammatory stimuli may originate from neurons, triggering secondary inflammatory responses mediated by astrocyte-microglia crosstalk to drive disease progression. Astrocyte-centric therapies require an understanding of how local and remote triggers integrate to induce diverse astrocyte response phenotypes.

### 2.3 Blood-brain barrier

The blood-brain barrier (BBB) is a crucial regulator of neuroimmune interactions, forming a dynamic interface consisting of brain microvascular endothelial cells, pericytes, neurons, astrocytes, and the extracellular matrix (26). Endothelial cells express tight junction proteins, solute carriers, and receptors to limit paracellular diffusion of water-soluble substances and to facilitate selective transport of nutrients and metabolites from blood to the brain. Under normal conditions, the BBB serves as a crucial physical barrier, restricting interactions between the peripheral immune system and the CNS (26) (**Figure 1C**). In pathological conditions involving BBB dysfunction, increased permeability may exacerbate neuroinflammatory responses due to immune cell infiltration and pro-inflammatory signaling. Nonetheless, acute neuroinflammation may play a crucial role in tissue repair and recovery (27), and understanding the molecular regulators of BBB-mediated neuroimmune interactions is crucial due to the context-dependent regulation of BBB integrity.

Tight junction proteins, such as claudin-5, occludin, ZO-1, and ZO-2, are essential for the function of the BBB. Germ-free (GF) or antibiotic-treated mice exhibit reduced expression of tight junction proteins and increased BBB permeability. Increased BBB permeability allows immunomodulatory blood components and immune cells to enter the CNS, promoting systemic immune-CNS interactions. Interventions like butyrate supplementation, early-life low-dose penicillin exposure to enhance SCFA-producing bacteria, or gut colonization with *Clostridium tyrobutyricum* or *Bacteroides thetaiotaomicron* can restore claudin-5 and occludin expression, thereby reinforcing BBB integrity to support CNS health.

### 2.4 Meningeal immunity

The meninges are the immunologically active barrier tissues that form the outer defense of the CNS. The dura mater, arachnoid mater, and pia mater form the multilayered meningeal structures that cover the CNS surface, crucial for immune surveillance, neuroinflammatory responses, and injury repair (27). Meningeal immune cells mainly consist of macrophage subtypes, along with various innate and adaptive immune cells, including dendritic cells, neutrophils, NK cells, T cells, B cells, and innate lymphoid cells. Located near the brain parenchyma, these immune cells release diverse pro- and anti-inflammatory cytokines that interact with receptors on neurons and glial cells (27). Meningeal immune cells, both resident and transient, can affect parenchymal cell function by reacting to peripheral signals.

The dural sinus system efficiently drains venous blood, while lymphatic vessels bridge the brain with cervical lymph nodes, facilitating immune communication. During localized inflammation, distinct meningeal layers coordinate precise immune cell recruitment and homing, with stromal cells acting as “commanders” to release regulatory factors for fine-tuned immune modulation. For example, γδ T cells are critical in regulating anxiety-like behaviors (28), whereas IgA^+^ plasma cells form a robust defense against fungal invasion. Specific T cell subsets, such as IFN-γ- (Th1) and IL-4-producing (Th2) cells, are essential for maintaining neuronal circuit stability and cognitive function (29). The meninges also serve as a niche for immature B cell development and selectively eliminate reactive B cells that threaten CNS integrity via apoptosis.

Growing evidence highlights the gut microbiome as a key determinant of meningeal immune function (30). The meningeal immune repertoire is replenished by circulating immune cells “trained” by the gut microbiota. Recent studies demonstrate that GF and antibiotic-treated mice exhibit impaired meningeal immune function due to reduced cell frequencies or suppressed secretory products. The gut microbiota influences meningeal humoral immunity, akin to the microbiome-guided development of IgA^+^ plasma cells in the gut (31). Reduced meningeal IgA^+^ B cells heighten CNS susceptibility to bloodborne pathogens. Colonization with specific pathogen-free (SPF) microbiota, *Citrobacter rodentium*, or segmented filamentous bacteria (SFB) restores IgA^+^ B cell levels, mitigating pathogen susceptibility (32). Suppressed IFN-γ expression in meningeal NK cells impairs astrocyte-mediated T cell apoptosis (via LAMP1 and TRAIL), weakening autoimmune control of the CNS (33, 34). Similarly, reduced IL-17a production by meningeal γδ17 T cells alleviates anxiety-like behaviors by limiting IL-17a signaling to cortical glutamatergic neurons (28).

In summary, the meninges and CNS border regions are emerging as critical hubs for gut-brain axis neuroimmune communication, though the functional diversity of their immune cell repertoire remains underexplored. Future studies should develop techniques to selectively manipulate and track meningeal immune subtypes to dissect their impact on neural function. Further investigation into the gut microbiome as a regulator of meningeal immunity may yield novel therapies targeting meningeal neuroimmune interactions for neurological disorders.

### 2.5 Microbiota-gut-brain axis

Similar to the BBB, the microbiome and gut mucosa form a barrier that critically shapes immune responses in the gut and distant organs (**Figure 1D**). As discussed, the gut microbiome is central to regulating microglial physiology, and targeting gut dysbiosis—common in neurological disorders—may restore microglial function. Reduced microbial diversity correlates with microglial deficits in morphology, maturation, activation, and pathogen responses, deficits which are reversible upon microbiota recolonization. GF mice exhibit increased BBB permeability, while restoring a pathogen-free microbiota normalizes BBB integrity. Microbiome abnormalities are linked to neurobehavioral disorders: early comparative studies revealed that GF mice display immature microglia with attenuated immune-related transcriptional programs (*e.g.*, type I interferon signaling, pathogen recognition, antigen presentation) but enhanced proliferation (Ki67, Ddit4) and survival (Csf1r, Pu.1) signals compared to SPF mice (35). Intriguingly, microglia from GF mice show upregulated expression of MAFB, a key transcription factor driving microglial maturation, suggesting developmental abnormalities may be microbiome-dependent (35). Consistent with this, four-week broad-spectrum antibiotic treatment (cefoxitin, gentamicin, metronidazole, vancomycin) in adult SPF mice replicates GF-like microglial immaturity, underscoring the necessity of continuous microbial signals for microglial homeostasis (35).

Beyond microbiome-BBB interactions in homeostasis, studies have evaluated microbial impacts on BBB integrity in neurological disease models. In experimental autoimmune encephalomyelitis (EAE), SPF microbiota transplantation corrects dysbiosis, reduces disease severity, and improves BBB function, as evidenced by reduced peripheral dye leakage into the brain and increased claudin-5 expression (36). In genetic hypertensive stroke models, cross-fostering with normotensive controls demonstrates that passive microbiota transfer enhances BBB integrity as measured via reduced IgG leakage and reverses stroke susceptibility (37). In murine models of traumatic brain injury (TBI), *Clostridium butyricum* and butyrate treatment restores BBB integrity by reversing the downregulation of occludin and zonula occludens-1 (ZO-1) observed following TBI (38). Probiotic combinations (e.g., *Bifidobacterium animalis lactis*, *Lactobacillus casei*) mitigate inflammation, improve BBB integrity, and enhance memory in aging and post-operative cognitive dysfunction models (39). Although mechanisms remain unclear, enhanced BBB integrity may limit systemic interactions of peripheral solutes, including antibodies, cytokines, and microbial metabolites, that may exacerbate neuroinflammation.

Emerging evidence suggests the microbiome may also regulate neuroprotective immune infiltration via BBB-independent pathways. Antibiotic-induced dysbiosis enhances BBB disruption (ZO-1/2 and occludin loss) while increasing monocyte infiltration in a CCR2-dependent manner (40). Conversely, probiotic mixture VSL#3 suppresses monocyte recruitment in inflammatory behavioral models (41). It should be noted that VSL#3 probiotic formulation after 2016 differs from the De Simone Formulation, which was commercially available under the trademark VSL#3^®^ until 2016 (42). These findings highlight microbiome-BBB crosstalk, but rigorous mechanistic studies are needed to dissect these interactions in health and disease.

### 2.6 Neural pathway regulation

Unlike cellular and humoral immune responses, neural pathways provide critical signaling mechanisms for gut-brain neuroimmune communication. Immune mediators and key cells, including microglia, endothelial cells, and astrocytes, play pivotal roles in brain development, plasticity, synaptic maintenance, and repair. Neuroinflammation is implicated in various diseases, including Parkinson’s disease, Alzheimer’s disease, and depression. Microbiome alterations, particularly in the gut, influence brain function and behavior, likely through neuroinflammatory mechanisms. The vagus nerve represents a crucial neuroimmune interface that mediates bidirectional communication between the gut and brain. When gut-derived lipopolysaccharide (LPS) from bacteria such as *Escherichia fergusonii* activates vagal afferent fibers through brainstem nucleus tractus solitarius (NST) neurons, it initiates a dual pathological cascade (43). First, LPS-stimulated vagal signaling upregulates hippocampal tumor necrosis factor-alpha (TNF-α) expression via α7 nicotinic acetylcholine receptor (α7nAChR)-dependent pathways, leading to a disruption in synaptic plasticity (44). Simultaneously, this process suppresses brain-derived neurotrophic factor (BDNF) production, impairing neurogenesis and memory consolidation (45). The pathway is further amplified by microbial tryptophan metabolites signaling through the aryl hydrocarbon receptor (AHR) in both vagal nerve terminals and hippocampal cells (46, 47). Importantly, surgical vagotomy completely abolishes these LPS-induced effects, confirming the vagus nerve’s essential role in this neuroimmune axis (48). This gut-brain communication pathway provides a compelling example of how peripheral immune signals can fundamentally reshape central nervous system functions.

The vagus nerve serves as a critical bidirectional communication pathway between the gut and the central nervous system. This neural conduit transmits various microbially-derived neuroactive compounds including γ-aminobutyric acid (GABA), serotonin (5-HT), dopamine, and acetylcholine (ACh), which activate specific receptors such as 5-HT3 receptors and interact with bacterial components including LPS and SCFAs. The gut microbiota actively modulates vagal nerve activity through multiple mechanisms: Toll-like receptors (TLRs) detect microbial products and activate nodose ganglia neurons, leading to serotonin release that subsequently influences vagal afferent fibers. This neural reflex circuit can exert either anti-inflammatory or pro-inflammatory effects on the gut microbiome. Importantly, intestinal inflammation creates a feedback loop by altering microbial composition, activating endotoxemia pathways, compromising intestinal barrier integrity, and facilitating bacterial product translocation - collectively reinforcing the bidirectional nature of gut-vagus interactions (**Figure 1E**).

From an efferent perspective, vagal motor neurons project to the intestinal wall where they release ACh to stimulate Brunner’s glands in the duodenal submucosa, promoting mucus secretion that supports commensal microbial colonization (**Figure 1F**). Vagal efferents also regulate intestinal absorption through mechanisms yet to be fully elucidated. During stress responses, sympathetic activation leads to norepinephrine (NE) release which: (i) modulates epithelial barrier function via α2A-adrenergic receptor (α2A-AR) signaling, and (ii) directly impacts microbial community dynamics. This complex neuroimmune-endocrine network exemplifies the sophisticated integration between the nervous system and gut function (**Figure 1E**).

## 3 Material basis of neuroimmune regulation in the MGBA

The nervous and immune systems both demonstrate adaptive plasticity and memory-like responses to external stimuli. While the nervous system reacts rapidly (within seconds) with localized signaling, the immune system exhibits slower but more widespread mobilization. Emerging evidence suggests that gut microbiota can influence astrocyte function through direct and indirect pathways; however, rigorous validation of these findings and deeper mechanistic insights into molecular and cellular interactions remain essential. Critical research priorities include characterizing the biological effects of microbial metabolites, such as indole derivatives that modulate cerebral AHR signaling, and deciphering the molecular mechanisms underlying microbiota-microglia/astrocyte crosstalk to mitigate neurological disorders. Structural components and metabolites derived from gut microorganisms are key mediators of immune regulation along the gut-brain axis. A comprehensive summary of recent advances in this field is provided in **Table 1**.

**Table 1:** MGBA-derived immunomodulators and their homeostatic control of host neuro-immunity.

Classification	Name	Sources		Potential main mechanism in the nerves immune system	References
Microbial components	LPS	Gram-negative bacteria	*Escherichia coli*	TLR4-NF-κB	(49)
	Outer membrane vesicles (OMVs)		*Bacteroides*	IFN-γ-MHC-II pathway	(50)
	LTA	Gram-positive bacteria	*Bacillus subtilis, Lactobacillus*	TLR2-Myd88	(51)
	Peptidoglycan		*Bifidobacterium, Lactobacillus*	NLRs	(52)
Microbial metabolites	SCFAs	Dietary fiber from *Bacteroides, Bifidobacteria, etc.*		Promote Treg differentiation and inhibit NF-κB pathway	(53)
	Tryptophan metabolites	Tryptophan metabolism from *Lactobacillus, Clostridium, etc.*		AhR signaling pathway activation	(46)

### 3.1 Short-chain fatty acids

SCFAs are the primary metabolites of the gut microbiota and play a central role in gut-brain axis signaling. These metabolites enter the brain via systemic circulation and lymphatic drainage, modulating immune and neurotransmitter activity to influence higher-order brain functions such as mood and cognition. SCFAs traverse the BBB to regulate microglial inflammatory responses, ameliorating neuroinflammation in Alzheimer’s disease (AD), autism, and Parkinson’s disease (PD). They also regulate neurotransmitter synthesis, exerting anxiolytic and antidepressant effects (**Table 1**). Major SCFA-producing gut bacteria include *Akkermansia muciniphila*, *Bacteroides*, *Bifidobacterium*, *Eubacterium*, *Streptococcus*, and *Lactobacillus*.

SCFAs are microbial fermentation byproducts of dietary fiber and potent modulators of host physiology. Mice treated with antibiotics and GF mice show significantly decreased SCFA levels, which are also associated with impaired microglial development (54). The SCFA butyrate also functions as a histone deacetylase inhibitor (HDACi) and enhances macrophage antimicrobial activity by suppressing HDAC3 (55). Acetate, the predominant short-chain fatty acid in the brain (56), also inhibits HDAC activity and expression while promoting histone hyperacetylation by acting as a substrate for histone acetyltransferases (57). Acetate supplementation counteracts LPS-induced H3K9 hypoacetylation and non-histone protein acetylation, thereby reducing inflammatory signaling in microglia (57). In rat models of neuroinflammation, acute acetate administration increases brain acetyl-CoA levels and decreases glial activation by 40–50% (58). Acetyl-CoA, a key metabolic intermediate in the TCA cycle and oxidative phosphorylation, is associated with macrophage polarization and neuroprotection (59). While further studies are needed to delineate direct vs. indirect SCFA effects on microglia *in vivo*, these findings suggest SCFAs modulate microglial function via epigenetic intermediates.

Clinical evidence shows dysbiosis in PD patients correlates with weakened SCFA signaling (60). In a 6-hydroxydopamine PD model, propionate supplementation improves motor function and reduces dopaminergic neuron loss. In AD models, butyrate restores synaptic plasticity, accompanied by reduced pro-inflammatory cytokine (TNF-α, IL-6, IL-1β) expression in the hippocampus and cortex (61). However, interventions targeting SCFA pathways in neurodegenerative mouse models yield inconsistent outcomes. These early studies suggest SCFA dysregulation may tilt the balance between neurotoxicity and neuroprotection by altering microglial function.

In summary, current evidence highlights the dominance of acetate, propionate, and butyrate (constituting 90% of total SCFAs) in key physiological processes, including modulation of intestinal pH, promotion of symbiont growth, suppression of appetite, lowering of cholesterol, reduction in fat storage, enhancement of gut barrier integrity, and mitigation of neuroinflammation. Specific microbial-metabolite interactions are summarized in **Table 2**.

**Table 2:** The interaction between gut microbiota and short-chain fatty acids has significant implications for maintaining immune homeostasis in the host nervous system.

SCFAs	Gut microbe	Potential roles in the nerves immune system	References
Acetic acid	*Akkermansia muciniphila*	Cytotoxic T-cells↑ INF-γ↑	(62)
	*Bacteroides thetaiotaomicron*		(63)
	*Barmesiella intestinihominis*		(64)
	*Bifidobacterium*		(65)
	*Blautia faecis*		(66)
	*Christensenella minuta*		(67)
	*Clostridium pasteurianum*		(68)
	*Collinsella tanakaei*		(69)
	*Enterocloster asparagiformis*		(70)
	*Enterococcus casseliflavus*		(71)
	*Eubacterium limosum & E. ramulus*		(72, 73)
	*Succinatimonas hippei*		(74)
Propionic acid	*Acidipropionibacterium acidipropionici*	Cytotoxic T-cells ↑ INF-γ ↑ Dendritic cells ↑ LPS induced IL-6 and IL-12p40↓ Chemokines (cytokines CXCL11, CXCL10, CXCL9, CCL5, CCL4, CCL3, CXCL9, CCL5, CCL4, CCL3) ↓	(75)
	*Akkermansia muciniphila*		(76)
	*Bacteroides thetaiotaomicron*		(77)
	*Dialister succinatiphilus*		(78)
	*Phascolarctobacterium succinatutens*		(79)
	*Roseburia inulinivorans*		(80)
	*Veillonella*		(81)
Butyric acid	*Agathobacter rectalis*	Cytotoxic T-cells ↑ INF-γ ↑ Dendritic cells ↑ LPS induced TNF-α, IL-6 and IL-12p40 ↓ Chemokines (cytokines CXCL11, CXCL10, CXCL9, CCL5, CCL4, CCL3, CXCL9, CCL5, CCL4, CCL3) ↓ Antimicrobial protein cathelicidin LL-37 ↑ T cell induced IL-10 ↑ T cell induced IL-17 ↓ NF-κB activation ↓	(82)
	*Anaerobutyricum hali*		(83)
	*Anaerostipes hadrus*		(84)
	*Butyricimonas synergistica*		(85)
	*Butyricimonas virosa*		(86)
	*Christensenella minuta*		(87, 88)
	*Clostridium pasteurianum*		(89)
	*Desulfovibrio*		(90)
	*Eubacterium limosum & E. ramulus*		(91–94)
	*Faecalibacterium prausnitzi*		(95)
	*Gemmiger fomicilis*		(96)
	*Lachnospira eligens*		(97)
	*Lactobacillus*		(98)
	*Pseudobutyrivibrio*		(99)
	*Roseburia intestinalis*		(100)
	*Streptococcus salivarius*		(101)

Butyric acid exerts profound immunomodulatory effects on both innate and adaptive branches of the immune system. While acetic acid and propionic acid demonstrate interactions with certain immunological components, their immunoregulatory pathways are substantially fewer in scope and complexity compared to butyrate. ↑, increased expression; ↓, decreased expression.

### 3.2 Lipopolysaccharides

In humans, Gram-negative bacteria comprise up to 47.5% of the fecal microbiota, with *Bacteroidetes* being the dominant phylum (1, 102). Notably, LPS derived from *Bacteroides* spp. (LPS-BS) exhibits significantly lower endotoxic activity compared to *Escherichia* coli-derived LPS (LPS-E) and may represent the predominant form of LPS in the human gut (103). Although LPS is widely used to induce pro-inflammatory microglial responses and exacerbate CNS disorders, the diversity of effects induced by LPS (priming versus tolerance) must be considered, as these depend on LPS molecular heterogeneity, dose, timing, route of administration, and contextual gene-environment interactions, injury, or disease states (104–106). Intriguingly, systemic LPS preconditioning activates CNS tolerance mechanisms, mitigating subsequent brain injury and neuroinflammation (107). LPS exerts protective effects against cryogenic brain injury via microglial TLR4 activation, suggesting LPS-induced tolerance may originate within the CNS. Repeated low-dose LPS exposure induces a neuroprotective phenotype in the C8-B4 microglial cell line and primary peritoneal macrophages, potentially mediated by TRIF signaling and epigenetic reprogramming (108). However, it remains unclear whether gut-derived LPS or diverse LPS molecules from Gram-negative commensals can elicit CNS immunomodulatory effects.

Toll-like receptors (TLRs), best known for their role in innate immunity, are widely expressed on multiple cell types. In the CNS, TLRs are expressed by neural stem cells, neurons, oligodendrocytes, astrocytes, and microglia, regulating neurodevelopmental, neuroplastic, and neurodegenerative processes (109). Surprisingly, SPF mice with global TLR2/3/4/7/9 deficiencies exhibit normal parenchymal microglial density, morphology, and maturation, indicating that gut microbiota do not regulate microglial development or maintenance via TLR signaling. Nevertheless, GF mice display microglial functional defects, including impaired TLR responsiveness. Compared to SPF mice, GF mice exhibit attenuated microglial innate immune responses (reduced cytokine/chemokine production) following systemic or intracerebral LPS administration.

Notably, oral supplementation studies mimicking gut LPS exposure demonstrate that dietary LPS-E modulates neural functions, including anxiety-like behavior (110) and taste responses (111). Dietary LPS from *Pantoea* agglomerans ameliorates high-fat diet-induced memory deficits linked to β-amyloid accumulation, potentially via enhanced microglial phagocytosis (112). In GF mice, two-week LPS-E supplementation fully activates microglial antigen presentation and protects against neurotropic mouse hepatitis virus (JHMV) infection (113). These effects are mediated by microglial TLR4 signaling, as mice with microglia-specific TLR4 deletion show blunted LPS priming and exacerbated JHMV pathology. Consistent with the microglia-specific role of TLR4, bone marrow transplantation from TLR4-deficient mice to wild-type recipients does not alter microglial responses to oral LPS or sustained protection against JHMV (113). Collectively, these findings indicate that gut microbiota regulate microglial function via direct LPS-TLR4 interactions.

While gut LPS-induced microglial priming protects against infections, chronic neuroinflammatory diseases may be exacerbated by aberrant microbial antigen exposure. Elevated blood endotoxin levels promote systemic inflammation and blood-brain barrier (BBB) disruption, exposing microglia to peripheral pro-inflammatory mediators and amplifying neuroinflammation (114). Indeed, elevated LPS levels are observed in patients with amyotrophic lateral sclerosis (ALS) (115), Alzheimer’s disease (AD) (116) and severe autism (117). In healthy volunteers, intravenous LPS enhances systemic inflammation, activates microglia, and induces sickness behavior (118). However, whether gut-derived endotoxin directly activates microglia to drive neuroinflammation remains unresolved.

### 3.3 Aromatic compounds

Aromatic amino acids such as phenylalanine and tyrosine are metabolized by gut microbiota into diverse aromatic compounds. For example, tyrosine is metabolized to 4-hydroxyphenylpyruvic acid, 4-hydroxyphenyllactic acid, 4-methylphenol (p-cresol), and 4-hydroxyphenylethanol (tyrosol), which serve as critical mediators of host-microbiota crosstalk. Neurotransmitters derived from phenylalanine/tyrosine (*e.g.*, dopamine, norepinephrine, melanin) and tryptophan (*e.g*., serotonin) exhibit pleiotropic roles in the gut-brain axis. A common microbial pathway for phenylalanine/tyrosine metabolism involves AAA aminotransferase-mediated transamination, yielding compounds such as 4-hydroxyphenylpyruvic acid (4H-PAA), 4-hydroxyphenyllactic acid, p-cresol, and tyrosol.

**Figure 2:**
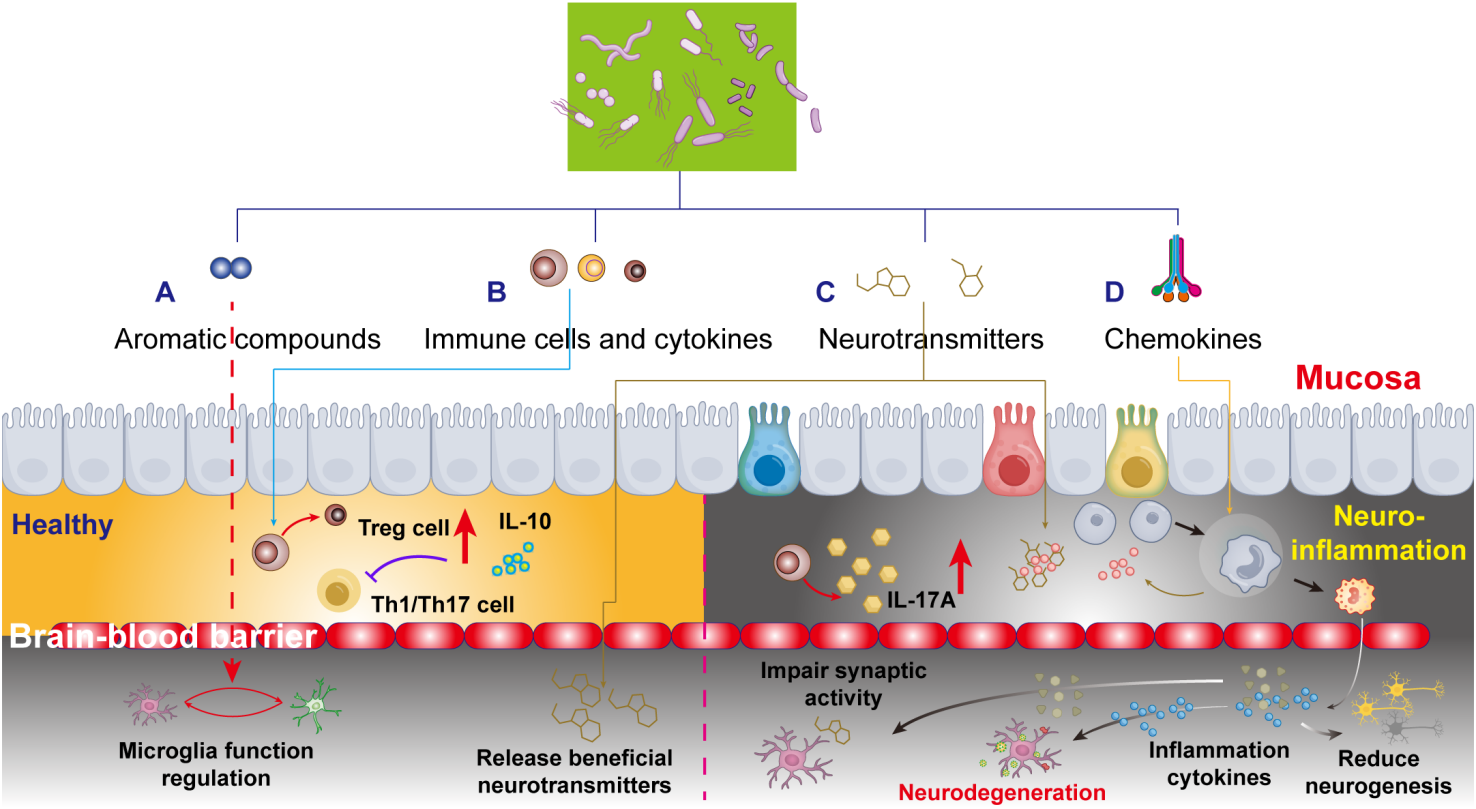
Schematic depiction of the microbiota-gut-brain axis and neuroinflammatory responses, emphasizing four key pathways by which aromatic compounds, cytokines, neurotransmitters, and gut chemokines interact with the blood-brain barrier (BBB) to influence brain function. **(A)** Aromatic compounds in healthy brain development. Mechanisms by which aromatic compounds promote brain development, emphasizing their regulatory roles in neurogenesis and neural circuit formation. **(B)** Gut-derived cytokines and neurodegeneration: functional alterations in gut immune cytokines and their impact on neurodegenerative pathologies, focusing on the secretory mechanisms of anti-inflammatory and pro-inflammatory cytokines. **(C)** Neurotransmitter regulation during brain development: the interplay between gut microbiota metabolites and neuronal function, revealing direct modulation of neural activity via microbial-derived neurotransmitters. **(D)** Gut chemokines in accelerated brain aging: pathological mechanisms linking gut chemokines to brain aging, including BBB permeability changes and neuroinflammatory cascades.

Tryptophan, an essential amino acid obtained solely from dietary sources, is another aromatic compound with significant neuroregulatory roles (**Table 1**, **Figure 2A**). Tryptophan metabolites, such as indoleacetic acid and indoleethanol, exhibit neuroprotective, antioxidant, and anti-inflammatory properties, and also regulate neurotransmission (e.g., serotonin). Tryptophan undergoes three major metabolic pathways in the gut: (a) microbial conversion to diverse indole derivatives; (b) serotonin (5-HT) synthesis via tryptophan hydroxylase in enterochromaffin cells; (c) degradation via the kynurenine pathway by indoleamine 2,3-dioxygenase 1 (IDO1) and tryptophan 2,3-dioxygenase (TDO) (119, 120). Notably, tryptophan acts as a key mood modulator and therapeutic target in cancer, autoimmune diseases, and neurological disorders (121, 122).

## 4 Potential mechanisms of MGBA in neuroinflammation

### 4.1 Microbiome-dependent T cells in multiple sclerosis

MS is a classical neuroinflammatory disorder characterized by disruption of the BBB, peripheral immune cell activation and infiltration, gliosis, and T cell-dependent demyelination. Its high heterogeneity arises from over 100 genetic susceptibility variants and environmental factors, such as vitamin D deficiency, circadian disruption, viral infections, and gut dysbiosis (123). Compared to healthy controls, the intestinal microbiome of patients with MS exhibits reduced abundances of *Prevotella*, *Faecalibacterium prausnitzii*, *Bacteroides coprophilus*, and *Bacteroides fragilis*, alongside elevated abundances of *Methanobrevibacter* and *Akkermansia muciniphila* (124). Impaired regulatory T cell (Treg) function, marked by reduced IL-10 secretion, diminishes their ability to suppress pro-inflammatory Th1/Th17-mediated neuroinflammation and contributing to the pathogenesis of MS (125). Clinically, MS patients show elevated levels of Th17 cells in cerebrospinal fluid (CSF) and the gut mucosa (126), characterized by increased IL-17a levels in serum and CSF (127) (**Figure 2B**).

### 4.2 Gut microbiota-associated neurotransmitters

Neurodegenerative pathophysiology involves dysregulation of neurotransmitter systems, including dopaminergic, cholinergic, serotonergic, glutamatergic, and GABAergic pathways. Critically, the gut microbiota modulates these systems to influence brain function (**Figure 2C**).

Serotonergic System: Dysregulation of serotonin (5-HT) signaling is implicated in Alzheimer’s disease (AD), affecting amyloid precursor protein (APP) processing and Aβ deposition. Patients with mild cognitive impairment (MCI) exhibit reduced availability of the brain serotonin transporter and higher cortical Aβ burden compared to controls. AD patients also show significantly lower urinary and serum serotonin levels. Notably, selective serotonin reuptake inhibitors (SSRIs) suppress Aβ levels in both human and AD mouse models.

GABAergic System: γ-Aminobutyric acid (GABA) is the primary inhibitory neurotransmitter. It is involved in regulating brain states, cognition (learning, memory, sensory processing), circadian rhythms, and motor function. Dysregulated tonic GABA currents are linked to Parkinson’s disease (PD) and Huntington’s disease (HD). The gut microbiota influences GABA production, and an overgrowth of *Escherichia* in patients with autism spectrum disorder (ASD) has been strongly correlated with aberrant GABA metabolism. Elevated GABA/glutamate (Glu) ratios serve as metabolic biomarkers for mild ASD.

Dopaminergic System: Microbiota-targeted therapies alleviate dopaminergic damage and motor deficits in PD models. Probiotic supplementation increases serum dopamine levels and improves motor function in PD patients. Berberine stimulates gut microbial production of L-DOPA and dopamine via tyrosine hydroxylase and DOPA decarboxylase activation, enabling dopamine synthesis in the brain.

Cholinergic System: Acetylcholine (ACh) is a key neurotransmitter modulated by the gut microbiota. For example, *Lactobacillus plantarum MTCC1325* restores ACh levels in a rat model of AD induced by D-galactose treatment by reducing acetylcholinesterase (AChE) activity. Similarly, prebiotic fructooligosaccharides counteract AChE elevation, rescuing cholinergic dysfunction in AD models.

### 4.3 AHR signal pathway

Tryptophan is an essential amino acid that is mainly obtained from the diet. The gut microbiota is crucial in regulating intestinal tryptophan metabolism and its key derivative pathways, such as kynurenine, serotonin, indole precursors, and aryl hydrocarbon receptor (AHR) ligands (128). Gut bacteria with tryptophanase convert dietary tryptophan into indole, which the host uses to synthesize AHR agonists like indoxyl sulfate and indole-3-propionic acid (IPA) (128). Notably, indoxyl sulfate and IPA are undetectable in GF mice, confirming that their bioavailability depends on the gut microbiota.

AHR is broadly expressed in both CNS-resident and peripheral immune cells, as extensively reviewed in the literature (129). In murine models of various diseases, including experimental autoimmune encephalomyelitis (129), ischemic stroke (130), intracerebral hemorrhage (131), and LPS-induced neuroinflammation (131), AHR expression is increased in various brain-resident cells, including microglia. However, the precise function of microglial AHR signaling in disease pathology is not yet fully understood. Systemic AHR deficiency exacerbates microglial activation in models of experimental autoimmune uveitis (132) and retinal degeneration (133), consistent with studies showing that microbiota-derived AHR ligands (e.g., urolithin A, indoxyl sulfate) suppress pro-inflammatory markers in microglia (134).

Recent studies, however, suggest dual pro- and anti-inflammatory roles for microglial AHR signaling. AHR silencing or activation via ligands (*e.g.*, formylindolo [3,2-b] carbazole or 3-methylcholanthrene) both inhibit LPS-induced microglial activation (135). This aligns with reports that AHR antagonists (6,2,4′-trimethoxyflavone) protect against ischemic stroke by blocking microglial activation and preserving neurogenesis (130, 136), while indoxyl sulfate promotes neurotoxic environments in glial co-cultures. Conditional AHR knockout in neural stem/progenitor cells attenuates astrogliosis and microgliosis in ischemic stroke models, whereas microglia- or astrocyte-specific AHR deletion worsens EAE (137). These findings indicate that AHR signaling mediates microbiota-neuroimmune crosstalk, with effects dependent on neuroinflammatory context, microbial agonist availability, and contributions from other AHR-expressing CNS/peripheral cells. The complexity of CNS AHR signaling underscores the importance of cell-cell interactions (*e.g.*, microglia-astrocyte crosstalk) during neuroinflammation.

In astrocytes, AHR signaling collaborates with the microbiota and neuroimmune networks to regulate neuroinflammation. Depleting AHR ligand-producing microbes with ampicillin weakens astrocytic AHR signaling, exacerbates EAE, and enhances NF-κB-driven pro-inflammatory gene transcription, leading to microglial activation (138). Similarly, microglia-specific AHR signaling suppresses astrocyte reactivity by modulating ligand ratios (TGF-α) for astrocyte receptors ERBB1 and FLT1 (137). Reduced TGF-α ratios in chronic MS lesions suggest dysregulated tryptophan metabolism and AHR signaling contribute to MS pathology. This aligns with findings that IPA inhibits LPS-induced inflammation in human astrocyte cultures (139), while TGF-α and VEGF-B suppress or amplify pro-inflammatory gene expression, respectively (137).

Clinically, potent synthetic AHR agonists (e.g., laquinimod) demonstrate therapeutic potential by targeting microbial tryptophan metabolism. In preclinical studies, laquinimod alleviates EAE symptoms and neuroinflammation via systemic and CNS immunomodulation (140, 141). Transplanting wild-type bone marrow into AHR-deficient mice partially reinstates laquinimod efficacy (140), independent of LysM+ immune cells (141). Astrocyte-specific AHR knockout significantly, but incompletely, blocks the effect of laquinimod (141). These results suggest optimal anti-inflammatory outcomes in CNS autoimmunity require systemic AHR signaling but highlight CNS-restricted AHR targeting (the primary site of EAE suppression) to minimize off-target effects.

### 4.4 Role of gut chemokines in neuroinflammation

The gut, as a highly innervated organ replete with immune cells, maintains a delicate equilibrium where integrity of the gut microbiota is essential for preserving intestinal barrier function. While systemic inflammation can compromise the BBB, oral antibiotic-induced dysbiosis further exacerbates BBB permeability through microbial metabolite-mediated mechanisms. Pathological conditions like “leaky gut” syndrome permit microbial byproducts and circulatory factors to stimulate peripheral immune cells and chemokine release, subsequently recruiting neutrophils, monocytes, and other inflammatory cells to affected sites. These chemokines orchestrate immune cell trafficking while dynamically regulating the functions of regulatory T cells (Tregs), type 3 innate lymphoid cells (ILC3s), and macrophages (131). The resultant cytokine milieu (including IL-10 and IL-22) profoundly influences neuronal viability, synaptic plasticity, and neurodegenerative processes (**Figure 2D**).

Chemokine-directed gut-to-brain immune cell migration operates through specialized molecular cascades that shape neurological disease progression. The CCL20-CCR6 axis exemplifies this mechanism, where intestinal epithelial-derived CCL20 guides ILC3s across compromised BBB regions into the CNS, where the production of neuroprotective IL-22 by the ILC3a mitigates demyelination in MS models (142, 143). In Alzheimer’s pathology, CXCR3^+^ immune cells facilitate the transport of gut-derived Aβ aggregates across both vascular and neural barriers, accelerating cerebral amyloidosis (144). Similarly, CCL2 overexpression disrupts BBB integrity in Parkinson’s disease, driving monocyte infiltration into the CNS while CCL5-mediated Th17 cell recruitment exacerbates dopaminergic neuron loss (145, 146). These chemokine networks not only regulate T cell differentiation and Th1/Th2 balance but also determine neuroinflammatory severity, as evidenced by the pivotal role of CCL2 in modulating the progression of MS through leukocyte trafficking control (147).

### 4.5 Gut microbiota dysbiosis in neurological disorders

The core neuroinflammatory mechanism induced by gut dysbiosis involves increased intestinal permeability allowing endotoxins (e.g., LPS) to enter circulation. This triggers pro-inflammatory cytokine release, subsequently disrupting the blood-brain barrier (BBB) and inducing neuroinflammation. In Alzheimer’s disease, acute enteritis may paradoxically reduce cerebral Aβ deposition (potentially via enhanced Aβ efflux into blood), but chronic dysbiosis interferes with Aβ clearance mechanisms, compromising its potential protective function as an antimicrobial peptide (148–150). Specific microbiota (e.g., *Klebsiella pneumoniae*) can invade the brain via the gut, exacerbating tau-mediated neurodegeneration in an ApoE genotype-dependent manner (151). Clinically, AD patients exhibit significantly reduced gut microbial diversity, with alterations in specific taxa (e.g., decreased *Ruminococcaceae*; increased *Bacteroidaceae*) correlating with cognitive impairment severity.

For Parkinson’s disease, dysbiosis-induced gut barrier damage promotes abnormal aggregation of misfolded α-synuclein (α-syn) in the enteric nervous system. This pathological protein undergoes retrograde transmission via the vagus nerve to the substantia nigra, forming Lewy bodies (152). Germ-free mouse models demonstrate that even with α-syn overexpression, the absence of gut microbiota completely prevents motor deficits and neuronal loss. In contrast, transplanting the microbiota from PD patients into wild-type mice induces α-syn aggregation and dopaminergic neuron death (153).

In MS, the specific eradication of the newly discovered gut bacterium *Erysipelotrichaceae OTU002* selectively reduces T cell activity, thereby diminishing the adhesion of myelin oligodendrocyte glycoprotein (MOG) to neuronal myelin and preventing the MS (154). Clinical studies indicate that microbial dysbiosis, characterized by an increase in *Alistipes*, is significantly correlated with worsened disability in MS, while elevated levels of short-chain fatty acid-producing bacteria such as *Eubacterium hallii*, *Butyricoccaceae*, and *Blautia* improve cognitive function and quality of life (155).

## 5 Summary and perspectives

This review introduces a novel perspective on the gut-neuroimmune axis by emphasizing the intricate mechanisms through which the gut microbiota influences the CNS. It goes beyond traditional views by delving into the specific roles of microbiota-derived components and metabolites in modulating the maturation and function of the BBB and CNS-resident immune and glial cells. By elucidating these molecular pathways, the study uncovers novel functional microbial species and effector molecules that have not been previously explored in depth. This in-depth exploration of the gut-brain connection not only advances our understanding of the microbiota’s role in neuroimmune regulation but also opens new avenues for innovative therapeutic interventions.

The clinical implications of this review are profound. By highlighting the key role of the gut microbiota in maintaining neuroimmune homeostasis, the study provides a theoretical foundation for the development of microbiota-based therapies for neurological disorders. By identifying specific microbial species and metabolites that regulate neuroimmune interactions, researchers can target these molecules to restore balance in the gut-brain axis, potentially mitigating the symptoms of diseases such as MS, PD, HD, and AD. This innovative approach holds the promise of more effective and personalized treatment options for patients, ultimately leading to improved quality of life and outcomes.
